# Trophic Structure in a Seabird Host-Parasite Food Web: Insights from Stable Isotope Analyses

**DOI:** 10.1371/journal.pone.0010454

**Published:** 2010-05-04

**Authors:** Elena Gómez-Díaz, Jacob González-Solís

**Affiliations:** Institut de Recerca de la Biodiversitat i Departament Biologia Animal (Vertebrats), Universitat de Barcelona, Barcelona, Spain; Institut Pluridisciplinaire Hubert Curien, France

## Abstract

Ecological studies on food webs rarely include parasites, partly due to the complexity and dimensionality of host-parasite interaction networks. Multiple co-occurring parasites can show different feeding strategies and thus lead to complex and cryptic trophic relationships, which are often difficult to disentangle by traditional methods. We analyzed stable isotope ratios of C (^13^C/^12^C, δ^13^C) and N (^15^N/^14^N, δ^15^N) of host and ectoparasite tissues to investigate trophic structure in 4 co-occurring ectoparasites: three lice and one flea species, on two closely related and spatially segregated seabird hosts (*Calonectris* shearwaters). δ^13^C isotopic signatures confirmed feathers as the main food resource for the three lice species and blood for the flea species. All ectoparasite species showed a significant enrichment in δ^15^N relatively to the host tissue consumed (discrimination factors ranged from 2 to 5‰ depending on the species). Isotopic differences were consistent across multiple host-ectoparasite locations, despite of some geographic variability in baseline isotopic levels. Our findings illustrate the influence of both ectoparasite and host trophic ecology in the isotopic structuring of the *Calonectris* ectoparasite community. This study highlights the potential of stable isotope analyses in disentangling the nature and complexity of trophic relationships in symbiotic systems.

## Introduction

Parasites have been by far the missing links in natural food webs partly due to the singularity and multidimensionality of parasitic interactions [1], [2]. Importantly, parasites often have complex life cycles with multiple hosts and feeding preferences so they can occupy several trophic levels, which may lead to intermingled and even cryptic host-parasite trophic relationships that can be difficult to disentangle [3]. Trophic studies in host-parasite networks are scarce and traditional approaches applied to characterize parasite feeding preferences and host use have important drawbacks or are of difficult endeavour in natural conditions [4]. These include for example behavioural and observational studies of parasite dietary breadth, laboratory experiments on parasite feeding preferences and direct examination of parasite's gut contents. But most of these methods are qualitative whereas the temporal integration they provide is usually very short [1], [5]. In this context, the use of indirect methods, such as biochemical markers, can expand our understanding of parasite trophic ecology and represent an alternative and innovative tool in host-parasite food-web studies [6].

Naturally occurring stable isotopes are widely used in food web studies as dietary tracers and to depict species' niches [7], [8], [9]. This approach is based on the fact that stable-isotope ratios of nitrogen (^15^N/^14^N, denoted as δ^15^N) and carbon (^13^C/^12^C, δ^13^C) are transformed from dietary sources to consumers in a predictable manner [10]. Nitrogen signatures show an increase in the isotopic ratio throughout the trophic levels, i.e., typically from 2.5‰ to 5‰ greater [11], [12]. Carbon signatures show little change with trophic level but can be very useful indicators of the dietary source of carbon [10], [13]. In the case of host-parasite systems, stable isotope analysis has been successfully applied to help disentangle host-parasite food-web interactions in a number of parasite groups (see review by [1]). In parasites, as consumers, a stepwise enrichment reflected by greater δ^15^N values is expected relative to their hosts [11], [13], [14], but due to the complexity of host-parasite interactions the isotopic niche of a parasite can vary depending upon the host-parasite system [15], [16]. Furthermore, the level of enrichment can vary in a single parasite among hosts [17], or among parasite taxa within hosts [18], [19]. While much work has been done on a single parasite and/or host species, for now only few studies have taken a multi-parasite approach to examine host-parasite trophic interactions, and to our knowledge none of them has examined multiple host and parasite species simultaneously.

Seabirds, as most birds, host a plethora of parasitic organisms and thus represent an interesting model for such studies [20]. These include a great number of arthropod ectoparasites with contrasting life-history traits and feeding strategies while they also differ in the type of ecological interaction they establish with their host: from mutualistic (e.g., feather mites, [21], [22]) to parasitic (e.g., fleas, [23]). In the present study, we applied stable isotope analysis of nitrogen and carbon to examine trophic host-ectoparasite interactions of three lice and one flea species on two closely related seabird taxa of the genus *Calonectris*. The use of a multi-specific approach on a model system including several ectoparasites can shed new light on within-host resource partitioning (e.g., blood or feathers) shaping ectoparasite trophic structure (i.e., ectoparasite-specific trophic niches). Further, in *Calonectris* shearwaters both between and within species spatial differences in isotopic signatures have been previously reported [24]. Here applied, as differences in dietary sources (host tissues) should be reflected in their consumers (ectoparasites) isotopic values, we would expect host geographic variability to be detectable across trophic levels in their ectoparasites. Based on this prediction, our main aims were to investigate: i) resource partitioning among ectoparasites within a host, and ii) how between-host and within-host species geographic variability can influence the isotopic structuring of the ectoparasites.

## Materials and Methods

### Ethics statement

All animals were handled in strict accordance with good animal practice as defined by the current European legislation, and all animal work was approved by the respective regional committees for scientific capture (Consejería de Medio Ambiente del Cabildo de Gran Canaria, Canary Is., Spain; Secretaria Regional do Ambiente da Região Autónoma dos Açores, Azores Is., Portugal; Govern Balear, Balearic Is., Spain, Dpto. de Ecología y Medio Ambiente, Diputación de Almeria, Almeria, Spain, Dpto. Medio Ambiente Murcia, Murcia, Spain).

### Study species and sampling area

The Scopoli's *Calonectris diomedea* and the Cory's shearwaters *C. borealis* are two closely related taxa until now considered subspecies of the same species *Calonectris diomedea*. The two taxa have mostly disjoint distributions, across the Mediterranean Sea and the NE Atlantic, respectively. Although their taxonomic status is still being debated, a recent study suggest the two taxa should be regarded as separate rather than a single species [25].

**Figure 1:**
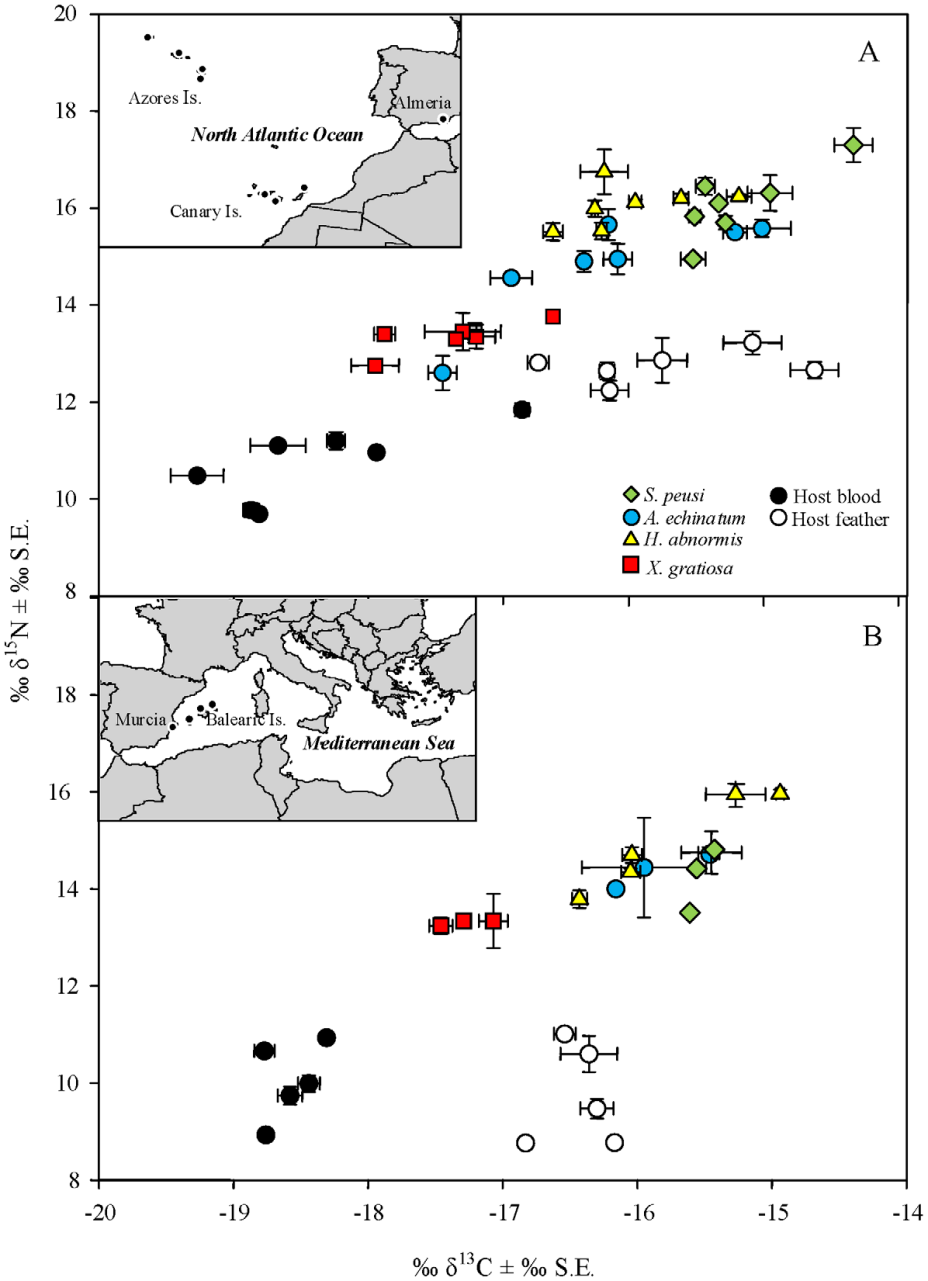
Isotopic composition of host and ectoparasite tissues across the study area. Geographic variation in mean δ^15^N and δ^13^C (± Standard Error) values of host tissue and ectoparasite species from 12 Atlantic (A) and Mediterranean (B) *Calonectris* breeding colonies.

The ectoparasite community of *Calonectris* shearwaters include three louse species: *Halipeurus abnormis* and *Saemundssonia peusi* (Ischnocera: Philopteridae), and *Austromenopon echinatum* (Amblycera: Menoponidae). One species of flea *Xenopsylla gratiosa* (Siphonaptera: Pulicidae), one species of soft tick *Ornithodoros maritimus* (Acarina, Ixodoidea, Argasidae) and, at least five different species of mites (Acari: Alloptidae and Avenzoariidae) [26]. During the breeding season, from 2003 to 2005 we collected ectoparasites, blood and the first primary feather from adult birds on 12 breeding colonies from three archipelagos (Balearic [4], Azores [4] and Canary Islands [2]), plus two off-coast islands in the SE of Spain (Almeria and Murcia) (Fig. 1). Not all described ectoparasites for *Calonectris* were present in all islands so further analyses were restricted only to lice and fleas. The prevalence and abundance of *Calonectris* ectoparasites varied as well among host individuals and breeding colonies [26], and in most cases not all four ectoparasite species co-occurred on a single host individual. Only adult parasites were included in the analysis and individuals of the same ectoparasite species and breeding colony were from different individual hosts. Details on the breeding sites, geographic coordinates and sampling sizes for each ectoparasite species and seabird host are specified in Table S1 (Supplementary material).

### Isotope analyses

Before isotopic analysis, ectoparasites and blood were dried in an oven at 60°C. Ectoparasites were identified to species, developmental stage and sex under a light microscope. Individual samples of each ectoparasite species were then weighted and placed into ultra-clean tin capsules. For some species, individual ectoparasites were pooled (2–3) to obtain the minimum mass for reliable stable isotope analysis. In those cases, each pool corresponded to a single host individual. Sample mass in ectoparasites ranged between 100–200 µg. Host feathers were washed in a 0.25 M sodium hydroxide solution, rinsed thoroughly in distilled water to remove any surface contamination, dried in an oven at 60°C to constant mass, and then grounded to powder using a SPEX 6750 Freezer/Mill. Host blood was dried and grounded to powder manually. To analyze stable isotopes, a sample mass equivalent to 0.375–0.400 mg of blood and feather powder was weighed and placed into ultra-clean tin capsule.

Samples were oxidized with CuO and CO_3_O_4_/Ag at about 900°C in a Flash EA 1112 Elemental Analyzer coupled to a pirolizator TC-EA and a breath bench, through an interface Conflo III Finnigan MAT. NO_X_ was reduced with Cu at 680°C. The combustion products, N_2_ and CO_2_, were dried using MgClO_4_ and transported to a Delta C Finnigan MAT mass spectrometer (Isotopic ratio mass spectrometry, Serveis Científico-Tècnics of University of Barcelona, Spain). Isotopic composition was expressed as the ratio of the heavy to light isotope relative to a standard, using delta (δ) notation: δX =  [(R_sample_/R_standard_)−1] ×1000, where X is ^13^C and ^15^N, R is the corresponding ration ^13^C/^12^C and ^15^N/^14^N, and the units are parts per thousand (‰). To monitor the accuracy of the results, international standards: IAEA CH_7_ (87% of C), IAEA CH_6_ (42% of C) and USGS 24 (100% of C) for ^13^C and IAEA N_1_ and IAEA N_2_ (with 21% of N) and IAEA NO_3_ (13.8% of N) for ^15^N, were ran each 12 samples to calibrate the system and compensate for any drift over time. Replicate assays of standard materials indicated measurement errors of ±0.1 and ±0.2 for carbon and nitrogen, respectively, but these are likely underestimates of true measurement error for complex organics like the ectoparasite tissues and feathers.

### Statistical analyses

Prior to building the models, we tested for a possible confounding effect of ectoparasite sex by comparing the δ^15^N and δ^13^C values between male and female individuals by ectoparasite species, by host taxa and by breeding colony, using univariate ANOVA. At the colony level, due to the limited sample size in some colonies, we restricted the analysis to 3 breeding localities (St. Maria, Lanzarote and Eivissa) (Table S1).

In the present study, ectoparasites were differentially distributed among geographic locations and not all four ectoparasite species were represented in all host breeding populations (see Table S1). To deal with this unbalanced design, we tested for differences in stable isotopic signatures among ectoparasite species and host tissues (blood and feathers) by applying a linear mixed model (LMM) using the restricted maximum likelihood (REML) estimation method. Geographic location was treated as a random term with the type of tissue (*H. abnormis*, *A. echinatum*, *S. peusi*, *X. gratiosa*, host blood and host feather) as a fixed factor. Considering geographic location as a random effect controls for possible pseudo-replication (i.e., multiple samples from the same site) and the noisy effect of site-to-site variation in sample sizes [27]. Using this model, we estimated the effect of the nature of the tissue, the percentage of the total variance due to geographic locality and whether this variance component differed significantly from zero (Wald Z-test). Bonferroni post-hoc comparisons among tissue types were performed to test for differences among ectoparasite species and host tissues.

To examine the extent of geographic variation in stable isotope signatures, we tested for differences in carbon and nitrogen mean isotope values among breeding localities and between regions in each ectoparasite species and host tissue using univariate ANOVA. Due to the limited sampled sizes for some colonies, we restricted the analysis to three colonies (St.Maria, Eivissa and Lanzarote), which cover most part of the breeding range of the host species and had the greatest sample size. Then, to examine whether ectoparasite isotope signatures co-vary relative to that of the host we examined isotopic similarity in ectoparasites and in host tissues (blood and feathers) across all breeding locations. First, we calculated dissimilarity matrices for each ectoparasite species and host tissues based on the Euclidean distances in mean carbon and nitrogen isotopic values among all pairwise combinations of breeding colonies. Then, to examine the correlation between host and ectoparasite spatial patterns of isotopic variation, we applied Mantel tests using the program zt [28]. The significance of the test was calculated by applying the permutation approach developed by Anderson and Legendre (1999) with 10,000 runs [29].

Discrimination factors of carbon and nitrogen were calculated as the difference between the isotopic ratios (δ^15^N or δ^13^C) in each ectoparasite species and the host tissue (blood or feather). Diet-tissue discrimination was denoted by *Δ^13^C* or *Δ^15^N host tissue- parasite* with specific subscripts to denote the host-tissue (blood or feather) and the ectoparasite species (*Ha*, *Ae*, *Sp* and *Xg*) [30]. Values reported are means ± standard deviation (Table 1). We then tested for differences in discrimination factors among ectoparasite species (mean colony values) using univariate ANOVA.

**Table 1:** Estimates of δ^13^C δ^15^N discrimination factors (Mean difference ± Standard deviation) between the host resource and the ectoparasite tissue for each ectoparasite species and breeding locality.

	Discrimination factor (‰)							
Island	*Δ^13^Cfeather-Ha*	Δ^15^N*feather-Ha*	*Δ^13^Cfeather-Ae*	Δ^15^N*feather-Ae*	*Δ^13^Cfeather-Sp*	Δ^15^N*feather-Sp*	*Δ^13^Cblood-Xg*	Δ^15^N*blood-Xg*
St.Maria	0.22	3.48	0.70	1.92	0.66	3.19	0.93	3.71
S.Miguel							0.92	2.98
Graciosa	0.42	3.28	0.06	2.71	0.61	2.71	0.59	2.34
Corvo	0.45	2.67			0.47	2.84	0.68	2.10
Lanzarote	0.56	3.58	0.60	2.84	0.29	4.64	0.23	1.92
G.Canaria	0.52	2.98	0.08	2.36	0.25	2.88	1.28	2.98
Almeria	0.42	3.19	0.52	2.86	1.72	3.51	1.02	2.23
***C.borealis***	**0.43±0.12**	**3.20±0.33**	**0.40±0.30**	**2.54±0.40**	**0.67±0.54**	**3.29±0.72**	**0.91±0.54**	**2.61±0.64**
Mallorca	1.36	6.49	0.84	5.27			1.51	3.61
Menorca	0.26	5.02			0.56	4.74		
Cabrera	1.56	7.19			1.38	5.99		
Eivissa	0.32	4.10	0.20	3.41	0.80	3.82	0.85	2.31
Murcia	0.49	3.34	0.59	3.44			1.48	2.68
***C.diomedea***	**0.80±0.62**	**5.23±1.61**	**0.54±0.32**	**4.04±1.07**	**0.91±0.42**	**4.85±1.09**	**1.28±0.37**	**2.87±0.67**
***Total***	***0.60±0.44***	***4.12±1.49***	***0.45±0.30***	***3.10±1.01***	***0.75±0.49***	***3.81±1.11***	***1.02±0.51***	***2.69±0.62***

All analyses, except the mantel test, were performed using SPSS 15.0 for Windows (IBM SPSS Statistics).

## Results

We analyzed the carbon and nitrogen stable isotope signatures of host blood and feathers as well as of whole individuals for each ectoparasite species to investigate geographic variation and trophic relationships of ectoparasites in relation to their hosts. We found significant differences in isotopic signatures among all four ectoparasite species and host tissues. In addition there was a spatial variation in ectoparasite and host isotopic values across geographic locations (Fig. 1, Table S1).

### Trophic structure

Sex was not significant for any ectoparasite species analyzed when pooled from different host taxa and breeding colony (*H. abnormis* (M:24, F:27): δ^15^N F_1,51_ = 0.126 P = 0.82, δ^13^C F_1,51_ = 0.235 P = 0.79; *A. echinatum* (M:13, F:15): δ^15^N F_1,27_ = 1.598 P = 0.22, δ^13^C F_1,27_ = 0.190 P = 0.67; *S. peusi* (M:14, F:14): δ^15^N F_1,27_ = 3.689 P = 0.07, δ^13^C F_1,27_ = 0.186 P = 0.67; *X. gratiosa* (M:20, F:20): δ^15^N F_1,39_ = 1.802 P = 0.82, δ^13^C F_1,39_ = 0.077 P = 0.78), nor when analyzed separately by host taxa or breeding colony (all P>0.05), thus further models were built pooling together males and females from individual hosts.

Results of the linear mixed model analysis showed that tissue type (blood, feather, the flea and the three lice) had a significant effect on both nitrogen (F_5, 241_ = 315.26, P<0.001) and carbon (F_5, 241_ = 3094.56, P<0.001) stable isotope signatures. In line with this, post-hoc Bonferroni adjusted pairwise comparisons indicated greater carbon and nitrogen isotopic signatures for lice than fleas (All P<0.0001). Among lice there were significant differences in nitrogen between body (*A. echinatum*; Family Amblycera) and wing lice (*Ha*: D = −0.82, P = 0.001, and *Sp*: D = −0.87, P = 0.003), but not between the two wing lice species (Family Ichnocera), which showed similar values (D = −0.05, P = 1.00). Overall, δ^15^N signatures of all four ectoparasites were greater than those of the host blood or feathers (All P<0.001). In the case of carbon comparisons among the three lice and the flea were all significant (All P<0.001) as well as between the two wing lice *H. abnormis* and *S. peusi* (D = −0.695, P<0.001). Similarly, there were significant differences between values of host blood and all other tissue types (All P<0.001), however significance did not hold in the case of host feathers which showed similar carbon values to lice except for the louse *S. peusi* (D = 0.136, P<0.001). Results of the LMM model showed that the percentage of variance explained by geographic location was 41.2% for nitrogen (Wald Z = 1.99, P = 0.046) and 29.9% for carbon (Wald Z = 2.15, P = 0.031). Although tissue type was the major factor affecting stable isotope values observed, these results suggest that breeding locality had a significant effect on patterns of isotopic variation, and this effect seemed to be stronger in the case of carbon.

**Figure 2:**
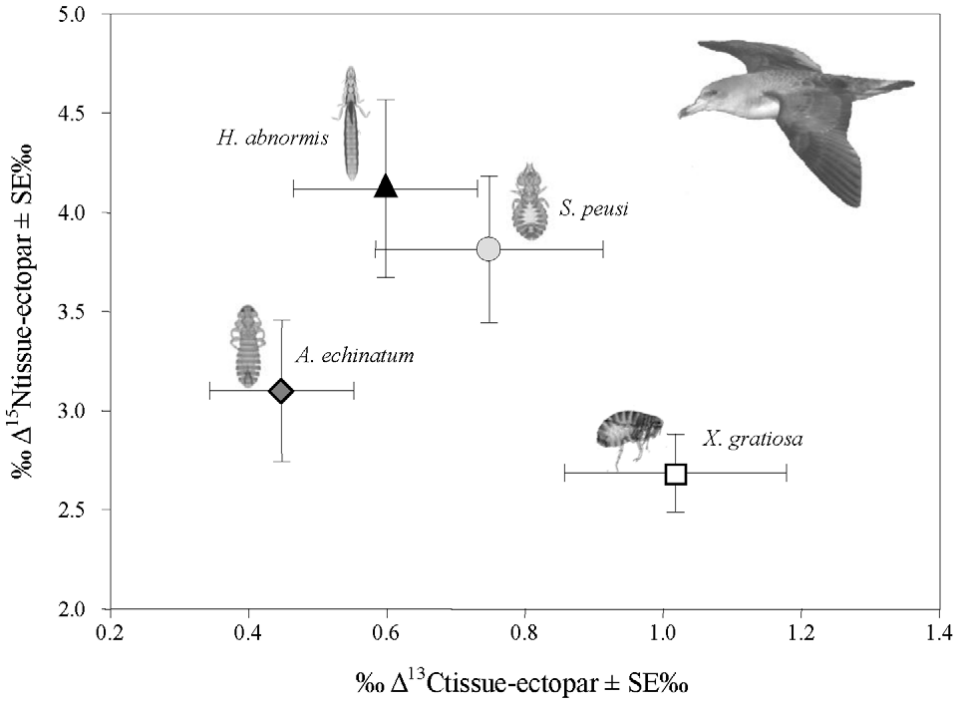
Isotopic enrichment in *Calonectris* ectoparasites. Mean Δ^15^N and Δ^13^C discrimination factors among ectoparasite tissues and host tissues (mean colony values ± Standard Error).

Finally we examined the enrichment, as indicated by the discrimination factor of each ectoparasite species relative to the host tissue consumed (i.e., mean values across multiple breeding sites) (Fig. 2, Table 1). All ectoparasite species were significantly enriched in both carbon and nitrogen isotope values relative to the host tissue consumed (t-test, all P<0.001). Nitrogen discrimination factors ranged from 2 to 5 ‰ depending on the ectoparasite species, host tissue type and breeding locality (Table 1), but was generally greater for lice (*Δ^15^Nfeather-Ha* = 4.12±1.49, *Δ^15^Nfeather-Ae* = 3.10±1.01, *Δ^15^Nfeather-Sp* = 3.81±1.11, *Δ^15^Nblood-Xg*: 2.69±0.62). On the contrary, lice appeared relatively low carbon enriched (0.45–0.75 ‰) compared to the flea species, which showed the greatest carbon discrimination factor (*Δ^13^Cblood-Xg*: 1.02±0.51) (Table 1). Overall, carbon and nitrogen discrimination factors differed among ectoparasite species (*Δ^15^N*: F_3,37_ = 3.45, P = 0.027; *Δ^13^C*: F_3,37_ = 2.73, P = 0.059) (Fig. 2). However, post-hoc Bonferroni tests indicated significant differences only among some lice and the flea species (pairwise comparisons: *Δ^15^N Ha-Δ^15^N Xg*: D = 1.43, P = 0.006; *Δ^13^C Ae-Δ^13^C Xg*: D = −0.57, P = 0.068).

Previous studies suggest variation in the elemental composition or isotopic ratio of host tissues can affect discrimination factors in the ectoparasites [31]. In the present study samples were not subjected to lipid or chitin extraction prior to analysis. We detected slight differences between the C/N ratios of the ectoparasites and host tissues (Table S1), therefore a potential effect of these host and ectoparasite specific compounds on carbon signatures of parasites cannot be completely ruled out.

### Geographic structure

To test for spatial differences in stable isotope signatures across the study area, we first restricted the analysis to three localities with greater samples sizes (St. Maria, Lanzarote and Eivissa; see above). We found significant differences in mean carbon and nitrogen isotope signatures of the host tissues among these breeding colonies (δ^15^N: tissue F_1,49_ = 21.32, P<0.001; colony F_2,44_ = 15.83, P<0.001; tissue* colony F_2,44_ = 7.76, P<0.001, δ^13^C: tissue F_1,44_ = 68.84, P<0.001; colony F_2,44_ = 25.35, P<0.001; tissue* colony F_2,44_ = 0.48, P = 0.627) (Fig. 1). However, in the case of nitrogen results showed a significant interaction between host tissue and breeding colony, indicating that among tissue differences in mean isotopic values differ among colonies. Similarly, stable isotope values of the ectoparasite species also differed among the three breeding localities tested (δ^15^N: ectoparasite F_1,49_ = 30.35, P<0.001; colony F_3,61_ = 14.01, P<0.001; ectoparasite* colony F_2,61_ = 1.70, P = 0.136, δ^13^C: ectoparasite F_3,61_ = 31.53, P<0.001; colony F_2,61_ = 23.86, P<0.001; ectoparasite* colony F_2,61_ = 0.85, P = 0.536), but in this case interactions were not significant. We then examined patters of co-variation in stable isotope signatures between host and ectoparasites across the study area using Mantel test. In all species, we found a significant relationship between ectoparasite and host tissue isotopic differences among colonies (i.e., Euclidean pairwise distances). Correlations and significance were greater when comparing the ectoparasite tissue with its consumed host tissue than when compared with another host tissue (Mantel tests: *Ha-feather* r_12_ = 0.51, P<0.001; *Ae-feather* r_12_ = 0.72, P<0.001; *Sp-feather* r_12_ = 0.68, P<0.001; *Xg-blood* r_12_ = 0.65, P<0.001).

## Discussion

### Trophic structure of *Calonectris* ectoparasites

*Calonectris* ectoparasites appeared both carbon and nitrogen isotopically enriched relative to the consumed tissue of their hosts. Despite some geographic variability, within each locality isotopic values of parasites showed a similar offset relative to the tissue consumed (blood or feather). Our results confirm the stepwise enrichment in ectoparasite nitrogen signatures as expected between a consumer and its diet (from 2.5‰ to 5‰) [14]. This result, however, contrast with some studies showing similar or even depleted nitrogen isotopic levels in parasites (see review by [1]). Explanations to this include the intrinsic characteristics of the hosts or parasites and the nature of the ecological interaction [1]. Interestingly, not only do *Calonectris* ectoparasites occupy a higher trophic level relative to their host, but there were substantial differences in the level of enrichment among species (from 2 to 5‰; Table 1, Fig. 2). Intra-host differences among parasite taxa have been previously reported [18], [19]. In this case, the level of enrichment can reflect parasite-specific preferences in the host resources they consume, i.e., resource partitioning [16]. Thus, we can make inferences regarding ectoparasite trophic niche by comparing ectoparasite isotopic signatures with those of the different host tissues (i.e., blood and feathers). For instance, the flea *X. gratiosa* showed about 1‰ enrichment in carbon and 2–3‰ enrichment in nitrogen signatures compared to the isotopic signatures of the host blood, thus pointing out its haematophagous diet. In contrast, all three lice species showed similar shifts in the enrichments in carbon and nitrogen signatures relative to those from host feathers, indicating their preferences on feathers remains and other debris. Nevertheless, some Ambliceran lice species can behave as facultative haematophages [32], while also feed on corporal secretions [33], [34], which may explain slightly different isotopic values among lice. Future studies including other types of host tissues, i.e., uropygial gland secretions or different feather types, may help in providing further details in the degree of trophic specialization among the different species of lice. Recent studies suggested the isotopic differences we found among ectoparasites may also arise from sources of variation others than diet (e.g., taxon, sex, developmental stage, tissue) [31]. In the present study male and female of all ectoparasite species did not differ in their isotopic signatures while all ectoparasites analyzed corresponded to the adult stage (see results). In addition, to avoid potential biases associated to differences in isotopic values among the sampled tissues, we performed isotopic analyses on the whole ectoparasite body. Nevertheless, other confounding effects due to taxon-specific fractionation factors or specific components such as host lipids or arthropod chitin, could not be completely ruled out.

A common assumption of studies examining host-symbiont interactions is that all symbiotic organisms are parasitic. From a trophic point of view parasitism can be considered as a special case of predation in which a parasite consumes a part of its prey (host). However, not all parasites fit this definition; some may compete for resources with their hosts (e.g., intestinal nematodes [16]), while in some cases they can be even considered commensals or mutualists (e.g., some detritivorous insects) [2]). For instance, Neilson et al. (2005) found significantly low nitrogen enrichment in cestodes of rabbits compared to other endoparasite taxa, suggesting that these organisms might behave as facultative parasites feeding on a minor fraction of the gut contents thus causing none or little damage to the host [18], [19]. Similarly, some bird-ectoparasites typically categorized as parasitic, such as feather mites, do not appear to deplete the host of any vital resource, and may rather be mutualistic or at least commensal [21], [35]. These ectoparasites feeding on body secretions, detritus and rests of feathers may benefit birds in terms of feather cleaning and plumage maintenance [35], [36]. In such cases, the isotopic analysis of ectoparasite and host tissues emerges as a powerful tool to elucidate the nature of cryptic symbiotic relationships [16].

### Geographic variation in ectoparasite isotope ratios

According to the “*consumer-diet*” prediction for isotope ratios geographic variation in carbon and nitrogen signatures among ectoparasites should reflect those of their hosts in a predictable manner [11], [13], [37], [38], [39]. In the present study, not only are different ectoparasite taxa isotopically structured within hosts, but we also documented spatial variability at inter and intra-specific level in both nitrogen and carbon isotope signatures. That is, stable isotope values co-vary consistently in ectoparasites in relation to the seabird host and this is true at different spatial scales. At regional level (i.e., Mediterranean and Atlantic basins), this variability is concordant with the geographically disjoint distribution of the two *Calonectris* hosts breeding on Atlantic and Mediterranean archipelagos. Within each basin, among breeding localities, we also found differences in carbon and nitrogen isotope values among both ectoparasites and host tissues, although to a lesser extent. Nevertheless, the heterogeneity in patterns of prevalence and abundance of *Calonectris* ectoparasites [26], lead to an unbalanced sampling design and therefore the observed spatial patterns need further confirmation. Future studies, with complete parasite sampling at the level of individual host and several host replicates per colony will help to verify our findings and may add further insights into the isotopic structure of parasites at the intraespecífic level (e.g., individual or temporal variation).

Geographic differences observed at various trophic levels can be due to host-specific differences in ecology and behaviour [17]. In *Calonectris* shearwaters, some differences in the feeding ecology among populations can be found [40], [41]. While nitrogen mainly reflects trophic level, ^13^C values are mainly used to determine sources of primary production in foodwebs [7], and thus can be good indicators of spatial variability in isotopic baseline levels [10]. Previous studies on *Calonectris* shearwaters showed some changes in carbon isotopic signatures across the breeding range of the species [24]. In this sense, habitat-specific differences in isotopic baseline levels may better explain the spatial pattern observed in this study as differences in carbon isotopic signatures across host-parasite trophic levels.

### Conclusion and implications

Overall our results show marked trophic and spatial structure in the four ectoparasite species of *Calonectris* shearwaters considered in this study. Trophically, both carbon and nitrogen signatures in ectoparasites appeared enriched in relation to the consumed host tissue but the degree of enrichment varied among species, thus indicating some resource partitioning among species within the host body. Spatially, geographic variation in isotopic signatures in parasites mostly matched those observed for their hosts, which in turn reflect spatial gradients in baseline isotopic signatures across the host distribution. Finally, this is the first study reporting trophic relationships in a bird-ectoparasite system and demonstrates the potential of stable isotope analyses for revealing complex and multiple trophic interactions in symbiotic systems. Furthermore, the multi-specific approach here applied provides further insight into the complexity and dimensionality of trophic host-parasite interactions while adding further evidence to food web studies in parasites.

## Supporting Information

Table S1 Isotopic composition of ectoparasite and host tissues. Stable isotopes signatures, δ13C, δ15N (‰) and C/N ratios (%), for all four ectoparasite species from different Calonectris breeding colonies and by host taxa. Values report mean and standard error. Isotopic values for blood and feathers of hosts are also indicated (Hblood and Hfeather, respectively).
(0.09 MB DOC)
