# Supplementary material for: Trophic Structure in a Seabird Host-Parasite Food Web: Insights from Stable Isotope Analyses
Source: PLoS One. 2010 May 4;5(5):e10454. doi: 10.1371/journal.pone.0010454 (PMC2864259; doi:10.1371/journal.pone.0010454)
Supplement: Table S1 — Isotopic composition of ectoparasite and host tissues. Stable isotopes signatures, δ13C, δ15N (‰) and C/N ratios (%), for all four ectoparasite species from different Calonectris breeding colonies and by host taxa. Values report mean and standard error. Isotopic values for blood and feathers of hosts are also indicated (Hblood and Hfeather, respectively). (0.09 MB DOC) [file pone.0010454.s001.doc]

Table S1. Stable isotopes signatures, δ^13^C, δ^15^N (‰) and C/N ratios (%), for all four ectoparasite species from different *Calonectris* breeding colonies and by host taxa. Values report mean and standard error. Isotopic values for blood and feathers of hosts are also indicated (Hblood and Hfeather, respectively).

|  |  | ***Halipeurus abnormis*** | | | | ***Austromenopon echinatum*** | | | | ***Saemundssonia peusi*** | | | | ***Xenopsylla gratiosa*** | | | |
| --- | --- | --- | --- | --- | --- | --- | --- | --- | --- | --- | --- | --- | --- | --- | --- | --- | --- |
| **Host** | **Island** | ***n*** | ***δ^13^C*** | ***δ^15^N*** | ***C/N*** | ***n*** | ***δ^13^C*** | ***δ^15^N*** | ***C/N*** | ***n*** | ***δ^13^C*** | ***δ^15^N*** | ***C/N*** | ***n*** | ***δ^13^C*** | ***δ^15^N*** | ***C/N*** |
|  |  |  |  |  |  |  |  |  |  |  |  |  |  |  |  |  |  |
| ***C. borealis*** | St.Maria | 10 | -16.02 ± 0.05 | 16.12 ± 0.06 | 3.43 ± 0.03 | 6 | -16.94 ± 0.16 | 14.56 ± 0.05 | 3.29 ± 0.28 | 11 | -15.58 ± 0.04 | 15.83 ± 0.08 | 3.29 ± 0.05 | 9 | -17.88 ± 0.08 | 13.40 ± 0.13 | 3.22 ± 0.03 |
|  | S.Miguel | 2 | -16.25 ± 0.18 | 16.75 ± 0.46 | 3.94 ± 0.09 | 2 | -16.40 | 14.90 ± 0.21 | 3.37 ± 0.02 | 2 | -15.50 ± 0.07 | 16.45 ± 0.18 | 3.78 ± 0.45 | 2 | -17.95 ± 0.18 | 12.75 ± 0.04 | 3.20 ± 0.02 |
|  | Graciosa | 8 | -16.63 ± 0.08 | 15.51 ± 0.18 | 3.50 ± 0.04 | 2 | -16.15 ± 0.11 | 14.95 ± 0.32 | 3.29 ± 0.03 | 3 | -15.59 ± 0.09 | 14.95 ± 0.07 | 3.15 ± 0.15 | 2 | -17.35 ± 0.04 | 13.30 ± 0.07 | 3.64 ± 0.28 |
|  | Corvo | 3 | -16.27 ± 0.04 | 15.53 ± 0.17 | 3.53 ± 0.03 |  |  |  |  | 2 | -15.35 ± 0.04 | 15.70 ± 0.14 | 3.23 ± 0.01 | 2 |  | 13.35 ± 0.25 | 3.11 ± 0.05 |
|  | G.Canaria | 4 | -15.68 ± 0.06 | 16.20 ± 0.09 | 3.58 ± 0.09 | 4 | -15.08 ± 0.21 | 15.50 ± 0.11 | 3.49 ± 0.10 | 1 | -15.40 | 16.10 | 3.90 | 2 | -17.30 ± 0.28 | 13.45 ± 0.39 | 3.96 ± 0.29 |
|  | Lanzarote | 8 | -15.25 ± 0.09 | 16.24 ± 0.05 | 3.61 ± 0.02 | 6 | -15.28 ± 0.09 | 15.66 ± 0.32 | 3.32 ± 0.02 | 2 | -14.40 ± 0.14 | 17.30 ± 0.35 | 3.37 ± 0.01 | 8 | -16.63 ± 0.04 | 13.76 ± 0.12 | 3.21 ± 0.07 |
|  | Almeria | 2 | -16.32 ± 0.02 | 15.99 ± 0.17 | 3.67 ± 0.01 | 2 | -16.22 ± 0.02 | 14.74 ± 0.12 | 3.54 ± 0.02 | 2 | -15.02 ± 0.17 | 16.31 ± 0.37 | 3.30 ± 0.18 | 2 | -17.21 ± 0.01 | 13.43 ± 0.20 | 3.31 ± 0.03 |
| ***Mean C.b*** |  | ***37*** | ***-15.99 ± 0.11*** | ***15.97 ± 0.13.*** | 3.56 ± 0.03 | ***24*** | ***-15.98 ± 0.21*** | ***14.97 ± 0.14*** | ***3.36 ± 0.08*** | ***22*** | ***-15.39 ± 0.10*** | ***15.93 ± 0.18*** | ***3.34 ± 0.06*** | ***27*** | ***-17.33 ± 0.13*** | ***13.45 ± 0.16*** | ***3.30 ± 0.05*** |
|  |  |  |  |  |  |  |  |  |  |  |  |  |  |  |  |  |  |
| ***C.diomedea*** | Mallorca | 2 | -14.94 ± 0.03 | 15.96 ± 0.09 | 3.55 ± 0.01 | 2 | -15.47 ± 0.08 | 14.00 ± 0.11 | 3.46 ± 0.06 |  |  |  |  | 2 | -17.07 ± 0.11 | 13.34 ± 0.56 | 3.30 ± 0.06 |
|  | Eivissa | 3 | -16.04 ± 0.07 | 14.70 ± 0.16 | 3.65 ± 0.01 | 2 | -16.16 ± 0.01 | 14.75 ± 0.44 | 3.44 ± 0.03 | 2 | -15.56 ± 0.02 | 14.42 ± 0.08 | 3.51 ± 0.05 | 4 | -17.46 ±0.09 | 13.24 ± 0.17 | 3.38 ± 0.03 |
|  | Cabrera | 2 | -15.27 ± 0.22 | 15.95 ± 0.26 | 3.59 ± 0.01 |  |  |  |  |  |  |  |  |  |  |  |  |
|  | Menorca | 2 | -16.43 ±0.06 | 13.79 ± 0.18 | 3.64 ± 0.00 |  |  |  |  | 1 | -15.61 | 13.51 | 3.19 |  |  |  |  |
|  | Murcia | 6 | -16.05 ± 0.07 | 14.35 ± 0.07 | 3.58 ± 0.04 | 2 | -15.95 ± 0.46 | 14.44 ± 1.03 | 3.34 ± 0.04 |  |  |  |  | 7 | -17.29 ±0.02 | 13.34 ± 0.03 | 3.14 ± 0.03 |
| ***Mean C.d*** |  | ***15*** | ***-15.84 ± 0.14*** | ***14.77 ± 0.22*** | ***3.60 ± 0.02*** | ***6*** | ***-15.86 ± 0.21*** | ***14.39 ± 0.40*** | ***3.42 ± 0.03*** | ***3*** | ***-15.5 ± 0.10*** | ***14.37 ± 0.30*** | ***3.42 ± 0.06*** | ***13*** | ***-17.31± 0.06*** | ***13.28 ± 0.12*** | ***3.23 ± 0.04*** |
|  |  |  |  |  |  |  |  |  |  |  |  |  |  |  |  |  |  |
| ***Total*** |  | ***52*** | ***-15.95 ± 0.09*** | ***15.65 ± 0.13*** | ***3.57 ± 0.02*** | ***30*** | ***-15.97 ± 0.17*** | ***14.98 ± 0.14*** | ***3.37 ± 0.07*** | ***28*** | ***-15.41 ± 0.08*** | ***15.65 ± 0.19*** | ***3.36 ± 0.05*** | ***41*** | ***-17.32 ± 0.09*** | ***13.40 ± 0.11*** | ***3.28 ± 0.04*** |

Table S1 (continued).

|  |  | ***Host blood*** | | | | ***Host feathers*** | | | |
| --- | --- | --- | --- | --- | --- | --- | --- | --- | --- |
| **Host** | **Island** | ***n*** | ***δ^13^C*** | ***δ^15^N*** | ***C/N*** | ***n*** | ***δ^13^C*** | ***δ^15^N*** | ***C/N*** |
|  |  |  |  |  |  |  |  |  |  |
| ***C. borealis*** | St.Maria | 17 | -18.81 ± 0.04 | 9.69 ± 0.04 | 3.28 ± 0.06 | 4 | -16.23 ± 0.06 | 12.64 ± 0.18 | 3.12 ± 0.01 |
|  | S.Miguel | 6 | -18.87 ± 0.06 | 9.77 ± 0.14 | 3.27 ± 0.02 |  |  |  |  |
|  | Graciosa | 8 | -17.94 ± 0.03 | 10.96 ± 0.03 | 3.18 ± 0.01 | 3 | -16.21 ± 0.14 | 12.24 ± 0.21 | 3.17 ± 0.02 |
|  | Corvo | 4 | -18.67 ± 0.21 | 11.10 ± 0.05 | 3.15 ± 0.02 | 3 | -15.82 ± 0.19 | 12.86 ± 0.47 | 3.15 ± 0.01 |
|  | G.Canaria | 4 | -19.27 ± 0.20 | 10.48 ± 0.07 | 3.17 ± 0.05 | 3 | -15.15 ± 0.22 | 13.22 ± 0.25 | 3.15 ± 0.04 |
|  | Lanzarote | 15 | -16.86 ± 0.05 | 11.84 ± 0.02 | 3.20 ± 0.01 | 7 | -14.69 ± 0.18 | 12.66 ± 0.18 | 3.15 ± 0.01 |
|  | Almeria | 3 | -18.24 ± 0.07 | 11.20 ± 0.18 | 3.22 ± 0.03 | 3 | -16.74 ± 0.08 | 12.81 ± 0.12 | 3.16 ± 0.02 |
| ***Mean C.b*** |  | ***57*** | ***-18.11 ± 0.13*** | ***10.68 ± 0.13*** | ***3.22 ± 0.02*** | ***23*** | ***-15.46 ± 0.23*** | ***12.70 ± 0.21*** | ***3.15 ± 0.01*** |
|  |  |  |  |  |  |  |  |  |  |
| ***C.diomedea*** | Mallorca | 3 | -18.58 ± 0.09 | 9.74 ± 0.18 | 3.16 ± 0.02 | 2 | -16.30 ± 0.12 | 9.47 ± 0.20 | 3.17 ± 0.02 |
|  | Eivissa | 5 | -18.31 ± 0.04 | 10.93 ± 0.06 |  | 2 | -16.36 ± 0.21 | 10.60 ± 0.37 | 3.16 ± 0.02 |
|  | Cabrera | 2 | -18.44 ± 0.08 | 9.99 ± 0.17 | 3.13 ± 0.02 | 1 | -16.83 | 8.76 | 3.15 |
|  | Menorca | 1 | -18.76 | 8.93 | 3.15 | 1 | -16.17 | 8.77 | 3.19 |
|  | Murcia | 7 | -18.77 ± 0.08 | 10.66 ± 0.02 | 3.33 ± 0.07 | 4 | -16.54 ± 0.08 | 11.01 ± 0.07 | 3.16 ± 0.01 |
| ***Mean C.d*** |  | ***18*** | ***-18.57 ± 0.09*** | ***10.41 ± 0.14*** | ***3.22 ± 0.04*** | ***10*** | ***-16.51 ± 0.08*** | ***10.77 ± 0.40*** | ***3.17 ± 0.01*** |
|  |  |  |  |  |  |  |  |  |  |
| ***Total*** |  | ***75*** | ***-18.22 ± 0.10*** | ***10.61 ± 0.10*** | ***3.22 ± 0.02*** | ***33*** | ***-15.87 ± 0.16*** | ***11.94 ± 0.26*** | ***3.16 ± 0.01*** |
